# Herbivory on the pedunculate oak along an urbanization gradient in Europe: Effects of impervious surface, local tree cover, and insect feeding guild

**DOI:** 10.1002/ece3.8709

**Published:** 2022-03-14

**Authors:** Elena Valdés‐Correcher, Anna Popova, Andrea Galmán, Andreas Prinzing, Andrey V. Selikhovkin, Andy G. Howe, Anna Mrazova, Anne‐Maïmiti Dulaurent, Arndt Hampe, Ayco Jerome Michel Tack, Christophe Bouget, Daniela Lupaștean, Deborah Harvey, Dmitry L. Musolin, Gábor L. Lövei, Giada Centenaro, Inge Van Halder, Jonas Hagge, Jovan Dobrosavljević, Juha‐Matti Pitkänen, Julia Koricheva, Katerina Sam, Luc Barbaro, Manuela Branco, Marco Ferrante, Maria Faticov, Markéta Tahadlová, Martin Gossner, Maxime Cauchoix, Michał Bogdziewicz, Mihai‐Leonard Duduman, Mikhail V. Kozlov, Mona C. Bjoern, Nikita A. Mamaev, Pilar Fernandez‐Conradi, Rebecca L. Thomas, Ross Wetherbee, Samantha Green, Slobodan Milanović, Xoaquín Moreira, Yannick Mellerin, Yasmine Kadiri, Bastien Castagneyrol

**Affiliations:** ^1^ Univ. Bordeaux INRAE BIOGECO Cestas France; ^2^ 54744 A. N. Severtsov Institute of Ecology and Evolution Russian Academy of Sciences Moscow Russia; ^3^ Institute of Biology/Geobotany and Botanical Garden Martin Luther University Halle‐Wittenberg Halle Germany; ^4^ 530625 German Centre for Integrative Biodiversity Research (iDiv) Halle‐Jena‐Leipzig Leipzig Germany; ^5^ Research Unit ECOBIO (Ecosystems, Biodiversity, Evolution) UMR 6553 University of Rennes/Centre National de la Recherche Scientifique Rennes France; ^6^ Department of Forest Protection, Wood Science and Game Management Saint Petersburg State Forest Technical University St. Petersburg Russia; ^7^ 4321 Department of Geosciences and Natural Resource Management University of Copenhagen Frederiksberg C Denmark; ^8^ Forest Industries Research Centre University of the Sunshine Coast Sippy Downs Australia; ^9^ Biology Centre of Czech Academy of Sciences Entomology Institute Ceske Budejovice Czech Republic; ^10^ Faculty of Science University of South Bohemia Ceske Budejovice Czech Republic; ^11^ UniLaSalle AGHYLE UP.2018.C101 SFR Condorcet FR CNRS 3417 Beauvais France; ^12^ 7675 Department of Ecology, Environment and Plant Sciences Stockholm University Stockholm Sweden; ^13^ Forest Ecosystems' Research Unit Biodiversity Team Domaine des Barres INRAE Nogent‐sur‐Vernisson France; ^14^ Applied Ecology Lab Forestry Faculty “Ștefan cel Mare” University of Suceava Suceava Romania; ^15^ Department of Biological Sciences Royal Holloway University of London Egham UK; ^16^ Department of Agroecology Flakkebjerg Research Centre Aarhus University Slagelse Denmark; ^17^ 7675 Department of Ecology, Environment and Plant Sciences Stockholm University Stockholm Sweden; ^18^ 545707 Forest Nature Conservation Northwest German Forest Research Institute Hann. Münden Germany; ^19^ Forest Nature Conservation Georg‐August‐University Göttingen Göttingen Germany; ^20^ 48269 Department of Forest Protection Faculty of Forestry University of Belgrade Belgrade Serbia; ^21^ Forest Health and Biodiversity Natural Resources Institute Finland (LUKE) Helsinki Finland; ^22^ 3835 Spatial Foodweb Ecology Group Department of Agricultural Sciences University of Helsinki Helsinki Finland; ^23^ Dynafor Univ. Toulouse INRAE Castanet‐Tolosan France; ^24^ CESCO, Museum national d'Histoire naturelle CNRS Sorbonne‐Univ. Paris France; ^25^ 37809 Centro de Estudos Florestais Instituto Superior de Agronomia Universidade de Lisboa Lisboa Portugal; ^26^ Azorean Biodiversity Group cE3c – Centre for Ecology, Evolution and Environmental Changes University of the Azores Azores Portugal; ^27^ Forest Entomology Swiss Federal Research Institute WSL Birmensdorf Switzerland; ^28^ Department of Environmental Systems Science Institute of Terrestrial Ecosystems ETH Zürich Zürich Switzerland; ^29^ Station d'Ecologie Théorique et Expérimentale du CNRS Moulis France; ^30^ 244706 Department of Systematic Zoology Faculty of Biology Adam Mickiewicz University Poznan Poland; ^31^ Laboratoire EcoSystèmes et Sociétés En Montagne INRAE Univ Grenoble Alpes Saint‐Martin‐d'Hères cedex France; ^32^ 8058 Department of Biology University of Turku Turku Finland; ^33^ UR629 Ecologie des Forêts Méditerranéennes (URFM) INRAE Avignon France; ^34^ 56625 Faculty of Environmental Sciences and Natural Resource Management Norwegian University of Life Sciences Aas Norway; ^35^ 2706 Centre for Agroecology, Water and Resilience Coventry University Coventry UK; ^36^ 48269 Department of Forest Protection and Wildlife Management Faculty of Forestry and Wood Technology Mendel University in Brno Brno Czech Republic; ^37^ Misión Biológica de Galicia (MBG‐CSIC) Pontevedra Spain

**Keywords:** citizen science, impervious surface, insect herbivory, leaf gallers, leaf miners, local canopy cover, *Quercus robur*

## Abstract

Urbanization is an important driver of the diversity and abundance of tree‐associated insect herbivores, but its consequences for insect herbivory are poorly understood. A likely source of variability among studies is the insufficient consideration of intra‐urban variability in forest cover. With the help of citizen scientists, we investigated the independent and interactive effects of local canopy cover and percentage of impervious surface on insect herbivory in the pedunculate oak (*Quercus robur* L.) throughout most of its geographic range in Europe. We found that the damage caused by chewing insect herbivores as well as the incidence of leaf‐mining and gall‐inducing herbivores consistently decreased with increasing impervious surface around focal oaks. Herbivory by chewing herbivores increased with increasing forest cover, regardless of impervious surface. In contrast, an increase in local canopy cover buffered the negative effect of impervious surface on leaf miners and strengthened its effect on gall inducers. These results show that—just like in non‐urban areas—plant–herbivore interactions in cities are structured by a complex set of interacting factors. This highlights that local habitat characteristics within cities have the potential to attenuate or modify the effect of impervious surfaces on biotic interactions.

## Graphical abstract

We investigated the independent and interactive effect of impervious surface and local canopy cover on insect herbivory on the pedunculate oak throughout most of its distribution in Europe through a citizen science project. We found that local canopy cover within cities has the capacity to mitigate the effect of impervious surface on biotic interactions, as it influences the effect of impervious surface on herbivores differently. Therefore, this study supports the importance of maintaining trees in urban areas as it may consequently contribute to the preservation of biodiversity of insect herbivores in urban areas.

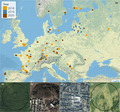

## 1 INTRODUCTION

Urbanization is one of the major drivers of global change (Grimmond, 2007; Ren, 2015). It causes fragmentation, isolation, and degradation of natural habitats (Pickett et al., 2001; Zipperer et al., 2000) in addition to creating warmer and drier conditions for both plants and animals (Chai et al., 2019; Taha, 1997; Wang et al., 2017). As a consequence, urbanization results in simplification of ecological communities and alteration of ecosystem processes, such as biotic interactions (Bang & Faeth, 2011; Fenoglio et al., 2020; Magura et al., 2010; McDonnell & Hahs, 2015). Understanding how the nature and strength of species interactions change along urbanization gradients contributes toward unravelling the mechanisms driving changes in species distribution and composition, which remain insufficiently known (but see Kozlov et al., 2015; Moreira et al., 2019; Turrini et al., 2016).

Plant–herbivore interactions play a pivotal role in ecosystems and consequently are one of the most studied biotic interactions (Jamieson et al., 2012; Stam et al., 2014). Analyses of insect herbivory patterns on woody and herbaceous plants along urban–rural gradients have received increasing attention in recent decades (Dreistadt et al., 1990; Kozlov et al., 2017; Moreira et al., 2019; Raupp et al., 2010). Several studies measured the response of a single herbivore species (Dale & Frank, 2014a; Long et al., 2019; Meineke et al., 2013; Parsons & Frank, 2019; Shrewsbury & Raupp, 2000; Turrini et al., 2016), responses of different herbivore feeding guilds (Cuevas‐Reyes et al., 2013; Kozlov et al., 2017; Moreira et al., 2019), or diversity and abundance of herbivores (Fenoglio et al., 2020; Rickman & Connor, 2003; Shrewsbury & Raupp, 2006; Youngsteadt et al., 2015) in urban compared to rural environments (but see Parsons & Frank, 2019). Although there seems to be a general tendency toward reduced insect abundance and diversity in urban settings compared to rural environments (Baldock, 2020; Blair & Launer, 1997; Fenoglio et al., 2020), there is no consensus on whether insect herbivory is higher (Christie & Hochuli, 2005; Moreira et al., 2019) or lower (Kozlov et al., 2017; Moreira et al., 2019; Nuckols & Connor, 1995) in urban compared to rural habitats. Given these mixed findings, a better understanding of the underlying ecological factors driving urbanization effects on insect herbivory is needed.

Several factors may explain the inconsistent effects of urbanization on insect herbivory reported in the literature. First, insect herbivore species vary markedly in their susceptibility to changing abiotic conditions (van der Putten et al., 2010; Zvereva & Kozlov, 2006) and might therefore exhibit different patterns of abundance and damage on focal host plants in urban *vs* rural areas (Kozlov et al., 2017; Moreira et al., 2019). In this sense, urban habitats are often associated with stressful climatic conditions (i.e., cities are warmer and drier than surrounding rural environments; Calfapietra et al., 2015; Dale & Frank, 2014b; Meineke & Frank, 2018) where endophagous herbivore guilds, e.g., leaf‐mining and leaf‐galling herbivores, could outperform exophagous herbivores, e.g., leaf chewers (Koricheva et al., 1998). Second, cities differ greatly in the amount of vegetation they harbor. The local tree cover (i.e., both overall tree density and potential host tree abundance) is a strong driver of urban biodiversity and trophic interactions between trees, insect herbivores, and their enemies (Herrmann et al., 2012; Long & Frank, 2020; Meyer et al., 2020; Stemmelen et al., 2020). More isolated trees frequently offer fewer resources to insect herbivores (Chávez‐Pesqueira et al., 2015), leading to a decrease in insect herbivory (Long & Frank, 2020). Isolated trees are also key (micro) habitats having a disproportionate importance for foraging predators, especially bats and birds (DeMars et al., 2010; Fischer et al., 2010; James Barth et al., 2015; Le Roux et al., 2018). At the same time, climatic conditions also vary with local tree cover resulting in high temperature and light intensity in more isolated trees, which may also influence insect herbivores (Dale & Frank, 2014b; Shrewsbury & Raupp, 2000). In this way, the amount and distribution of green areas—and in particular that of trees—could interfere with the effect of urbanization on leaf herbivory. Thus, the relative importance of these explanatory mechanisms needs to be confirmed along an urbanization gradient ranging from ‘green islands’ with high tree density to almost fully paved areas with only a few isolated trees.

In this study, we investigated the independent and interactive effects of impervious surface and local canopy cover on insect herbivory on the pedunculate oak (*Quercus robur* Linnaeus, 1753) throughout most of its geographic range in Europe. To this end, we quantified herbivory as the proportion of leaf area consumed or impacted by chewing and leaf‐mining herbivores as well as the incidence of leaf‐mining and gall‐inducing herbivores in leaf samples collected by professional scientists and schoolchildren in European countries between 2018 and 2020. We specifically predicted that: (a) insect herbivory decreases with impervious surface and increases with canopy cover; (b) the effects of impervious surface and canopy cover on leaf herbivory vary among the herbivore guilds; and (c) impervious surface and local canopy cover have an interactive effect on insect herbivory that vary among herbivore guilds. Overall, this work provides one of the most comprehensive studies to test for effects of impervious surface on plant–herbivore interactions and shed light on potential mechanisms underlying such effects.

## 2 MATERIALS AND METHODS

### 2.1 Study system

The pedunculate oak is one of the most common dominant deciduous tree species in European forests. It is also a popular ornamental tree in European urban areas (Eaton et al., 2016). Its distribution range spans from central Spain (23°N) to southern Fennoscandia (63°N) (Eaton et al., 2016). *Quercus robur* is associated with a large community of generalist and specialist herbivorous insects belonging to different feeding guilds (chewers, gall inducers, leaf miners, suckers, and xylophagous) (Marković & Stojanović, 2011; Moreira et al., 2018; Southwood et al., 2005). These ecological characteristics make the pedunculate oak a suitable object for measuring the effects of impervious surface and forest cover on plant–herbivore interactions.

### 2.2 Sampling network

**FIGURE 1:**
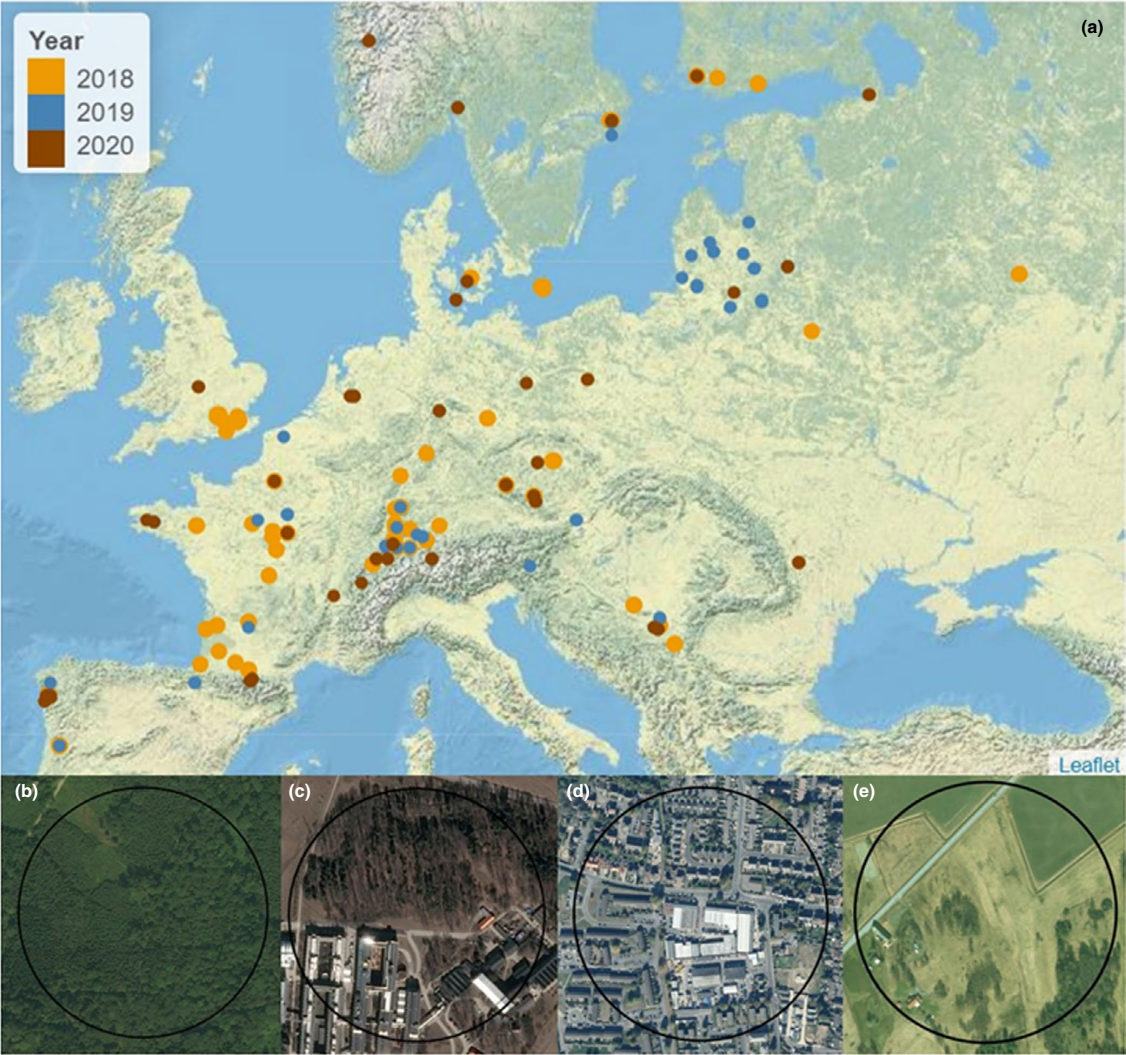
A map showing the location of trees sampled in 2018 (yellow circles), 2019 (blue circles), and 2020 (brown circles) by scientists and partner schools (a). Panels b–e show examples of 200 m radius buffers centered on sampled oak trees, with varying percentages of local canopy cover (in a buffer of 20 m radius) and impervious surface (in a buffer of 200 m radius). Panel b has 100% of local canopy cover and 0% of impervious surface; panel c has 50% of local canopy cover and 40% of impervious surface; panel d has 5% of local canopy cover and 65% of impervious surface; and panel e has 30% of local canopy cover and 10% of impervious surface. An interactive version of this map (a) is also included in the supplementary material as Figure S1. The aerial images (b, c, d, and e) are based on images from Bing maps 2021 and the map (a) was produced using Leaflet (Cheng et al., 2021)

The present study is a part of an ongoing citizen science project that involves to date a total of 93 participants, including 41 scientists and 52 school teachers and their classes from 17 European countries (Castagneyrol et al., 2019; Valdés‐Correcher et al., 2021), thereby covering most of the native geographical range of the pedunculate oak (Figure 1a). Partner scientists were instructed to sample three oaks in a wood or forest larger than 1 ha (which included large forests as well as small urban and peri‐urban forests), whereas schoolteachers were free to select one oak at their convenience. No particular criteria drove oak selection, except that trees had branches within easy reach from the ground and were reproductive (i.e., productive acorns). Subsequently, the dataset was mostly opportunistic in terms of the environments in which the oak trees were found, which included schoolyards, streets, parks, urban, and rural forests. We did not attempt to precisely characterize the surroundings of selected oaks.

We surveyed and geolocalized a total of 298 reproductive oak trees in forest and urban areas during 2018 (*n* = 132), 2019 (*n* = 56), and 2020 (*n* = 113), three of them been sampled twice in 2019 and 2020 (Figure 1). Pairwise distances between any two oak trees within a given site ranged from 4 to 2,359 m and was on average 185 m (median: 68 m), whereas the distance between the sites ranged from 947 to 3,696,375 m and was on average 629,770 m (median: 946,733 m). Scientists and schoolchildren applied the same protocol (Castagneyrol et al., 2019) to collect leaf material, from which herbivory data were acquired by one observer (YK, see below).

### 2.3 Leaf herbivory

In early summer (about 10–12 weeks after oak budburst at each location), scientists and partner schools haphazardly selected four low hanging branches per tree facing approximately compass directions. They haphazardly collected 30 leaves per branch (total: 120 leaves per tree). Then, 60 leaves were drawn blindly to reduce unconscious bias in leaf sampling. Scientists oven‐dried leaves for at least 48 h at 45°C right after collection (*n* = 203 oaks). Leaves collected by partner schools (*n* = 98 oaks) were oven‐dried when received by the project coordinators, to warrant optimal preservation prior to herbivory assessment (see Castagneyrol et al., 2019).

Three response variables were used to characterize leaf herbivory (Valdés‐Correcher et al., 2021): leaf damage (i.e., the percentage of leaf area removed or impacted by herbivores, including chewing and leaf‐mining herbivores), leaf‐miner incidence (i.e., the proportion of leaves with leaf‐mines), and leaf‐gall incidence (i.e., the proportion of leaves with galls). Herbivory was visually scored by assigning each leaf to one of the following classes: 0, 0.1–5.0, 5.1–10.0, 10.1–15.0, 15.1–25.0, 25.1–50.0, 50.1–75.0, or >75%, where the percentage represented the proportion of leaf surface removed by chewing herbivores or mined by leaf miners. We then used the midpoint of each class to average herbivory at the tree level (see Valdés‐Correcher et al., 2021 for details). We did not assess damage caused by sucking insects because punctures vary widely among species and for some species are not very visible (Schaefer & Panizzi, 2000). To minimize unconscious bias, herbivory was scored by a single trained observer (YK) who was unaware of leaf origin.

### 2.4 Landscape characteristics and climatic data

We defined the degree of impervious surface around focal trees as the percentage of impervious surface (including roads and buildings) in a buffer with a radius of 200 m centered on the focal oaks based on oak coordinates as retrieved from Google Maps by project partners (Meyer et al., 2020; Parsons & Frank, 2019). We chose the distance of a buffer of 200 m based on previous estimates of insect herbivore dispersal on the pedunculate oak (Barr et al., 2021; Zheng et al., 2015). We also calculated the percentage of local canopy cover within a 20 m buffer (excluding open areas and grasslands, and including the focal tree). We used this buffer size of local canopy cover because the local abundance of trees is a strong driver of urban biodiversity (Herrmann et al., 2012; Long & Frank, 2020; Meyer et al., 2020; Parsons & Frank, 2019; Stemmelen et al., 2020). To that aim we used the High Resolution Layers of the CORINE land cover datasets with 10‐m resolution and with reference year 2018 (±1 year) (Cover, 2018). Tree Cover Density extracted from the CORINE dataset ranges from 0 to 100%, while the impervious surface extracted from the CORINE dataset consists of artificially sealed areas (imperviousness ranging from 0 to 100%); variables were obtained using *R* 4.0.5 (R Core Team, 2020). We assumed that landscape characteristics did not change during the survey period (2018–2020).

To control for variability in herbivory that is influenced by local climatic conditions (Valdés‐Correcher et al., 2021), we extracted spring temperature and precipitation (mean temperature and precipitation in April–June) data from the WorldClim database with a spatial resolution of 5 min about 9 km at the equator (Fick & Hijmans, 2017) on the basis of the oak coordinates. Spring temperature and precipitation correspond to the period when most of the partners collected the leaves and also the main period of activity of insect herbivores on oak. Impervious surface and local canopy cover were slightly negatively correlated (Pearson *r* = −.38, *p* < .001, *n* = 298 trees), and were independent of latitude (Impervious surface: Pearson *r* = .02, *p* = .709; Local canopy cover: Pearson *r* = .04, *p* = .482) and climate (temperature and impervious surface: Pearson *r* = −.02, *p* = .800; temperature and local canopy cover: Pearson *r* = −.12, *p* = .037; Precipitation and impervious surface: Pearson *r* = .03, *p* = .594; Precipitation and local canopy cover: Pearson *r* = .01, *p* = .876). Although latitude was negatively correlated with temperature (Pearson *r* = −.76, *p* < .001) and precipitation (Pearson *r* = −.70, *p* < .001) which could have caused collinearity issue, a previous study found that climatic variables were better predictors of variation in herbivory, and therefore we decided to only include climatic variables in the models (Valdés‐Correcher et al., 2021).

### 2.5 Statistical analysis

All analyses were conducted in the *R* 4.0.5 (R Core Team, 2020) with packages *MuMIn* (*model*.*avg* and *dredge* functions) (Bartoń, 2020) and *lme4* (*lmer* and *glmer* functions) (Bates et al., 2018). We analyzed each of the response variables separately with generalized linear mixed‐effects models. We tested the effects of impervious surface, local canopy cover and their interaction, climatic variables, and year of sampling on leaf damage with Gaussian error distribution and identity link (the results were the same with a beta‐distribution and log‐link), and on the incidence of leaf miners and gall inducers with binomial error distribution and logit‐link in separate models. The data were not overdispersed, visual inspection of raw data did not call for zero‐inflated models, and the distribution of residuals met model assumptions.

In each model, Impervious surface (%), Local canopy cover (%), Impervious surface ×Local canopy cover, Year (as a factor), Spring temperature (°C), and Spring precipitation (mm) were included as fixed effects; and Partner ID as a random factor to account for the fact that some partners surveyed multiple trees and/or several years (note that each tree was only sampled once, and we thus did not account for Tree ID in the models).

We analyzed the data in the framework of information theory (Burnham & Anderson, 2002). We first built three models, one for each response variable separately (leaf damage, gall‐inducer, and leaf‐miner incidences). We scaled and centered all continuous predictor variables prior to modelling to make their coefficients comparable, and verified that uncontrolled correlations among explanatory variables were unlikely to bias model coefficient parameter estimates (all variance inflation factors lower than 2) (Zuur et al., 2009). We then applied a procedure of parsimonious model selection based on the Akaike's Information Criterion corrected for small sample sizes (AICc) and considered every model in a range of 2 units of AICc to the best model as equally likely (Arnold, 2010). We calculated the AIC weight (*w_i_*)—i.e., the probability that a given model is the best model within the set of candidate models—and also the relative variable importance (RVI), which reflects the importance of a particular variable in relation to all other variables, as the sum of *w_i_* of every model including this variable. When multiple models were competing with the best model (i.e., when several models with ΔAICc <2), we implemented a multi‐model inference approach, constructing a consensus model that comprised the selected variables from the set of best models. We subsequently averaged their effect sizes over all models in the set of best models, utilizing *w_i_* as the weighting parameter (i.e., model averaging). A certain predictor was deemed to have a statistically significant effect on the response variable if its 95% confidence interval (CI) did not bracket zero (Koricheva et al., 2013).

## 3 RESULTS

Impervious surface in a buffer of 200 m radius was on average 9.72 ± 0.91% (±SE, *n* = 298 trees) and ranged from 0 to 70%. Local canopy cover in a buffer of 20 m radius centered on focal oaks was on average 46.51 ± 1.90% and ranged from 0 to 100% cover.

**FIGURE 2:**
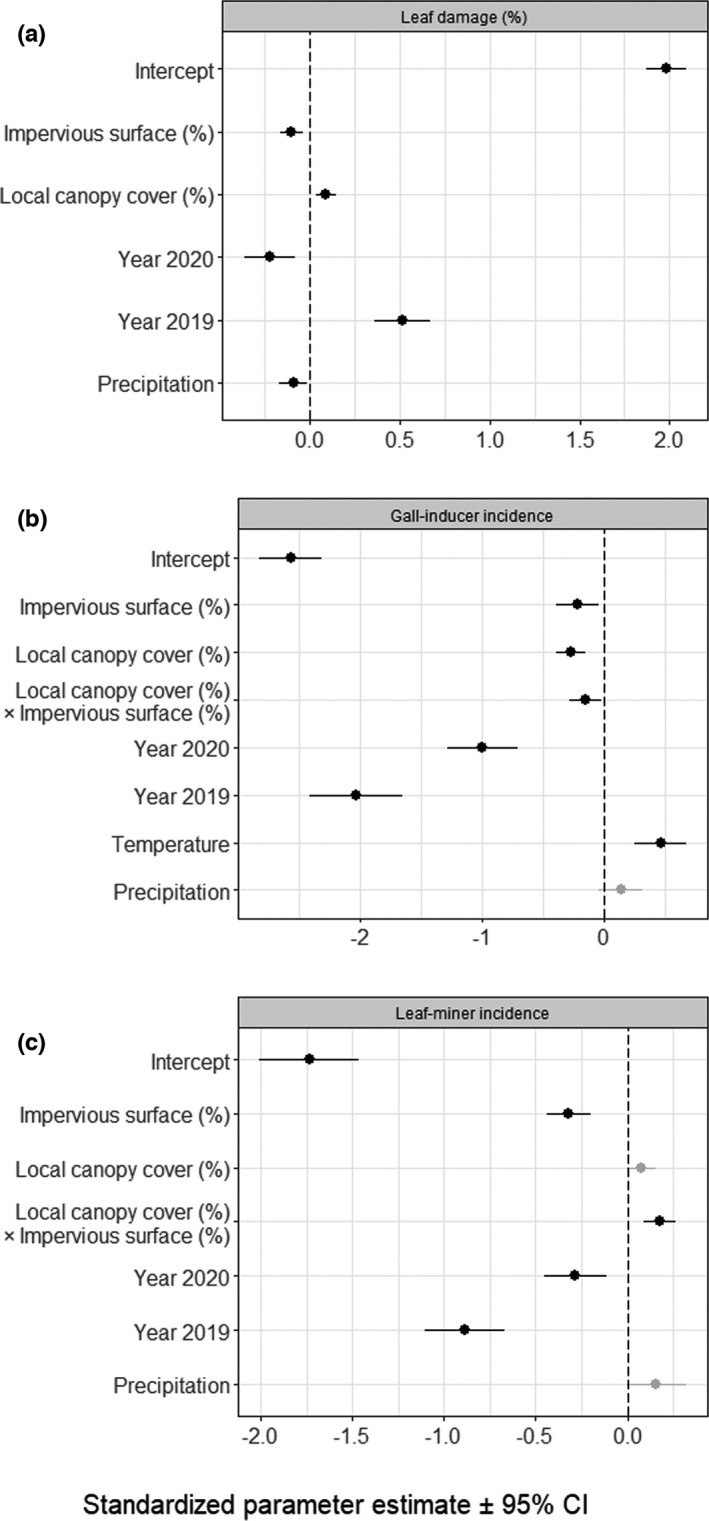
Standardized parameter estimates averaged across the best competing models testing the effects of percentage of impervious surface and local canopy cover, their interaction, year, mean spring temperature and/or mean spring precipitation (*n* = 298) on leaf damage (a), the incidence of gall‐inducing (b), and leaf‐mining (c) herbivores. Circles and error bars represent standardized parameter estimates and corresponding 95% CI. The vertical dashed line centered on zero indicates the null hypothesis. Black and grey circles indicate significant and non‐significant effect sizes, respectively. The year 2018 is the intercept and was contrasted with the years 2019 and 2020

Leaf damage was on average 7.72 ± 0.33% (17,880 leaves). Model selection retained models that included the percentage of impervious surface and local canopy cover, year, and spring precipitation as predictors explaining variability in leaf damage (Figure 2a, Table S1). Although model coefficient parameters averaged across the range of competing best models (i.e., with Δ AICc <2) were statistically significantly different from zero (Figure 2a), the relative importance of the variables retained was low, with the exception of the effect of the sampling year (Figure S2A). However, there was no clear threshold to decide whether a variable is important or not. Temperature was not retained and had a low relative importance (RVI < 0.25, Figure S2A). Specifically, leaf damage significantly decreased with increasing impervious surface (from 8.23 to 5.59% along the range of impervious surface, Figure S3A) and increased with local canopy cover (from 7.16 to 8.71% along the range of local canopy cover, Figure S3D). Leaf damage varied across years and was significantly greater in 2019 and lower in 2020 as compared to 2018 (Figure 2a). Leaf damage decreased significantly with increasing spring precipitation (Figure 2a).

**FIGURE 3:**
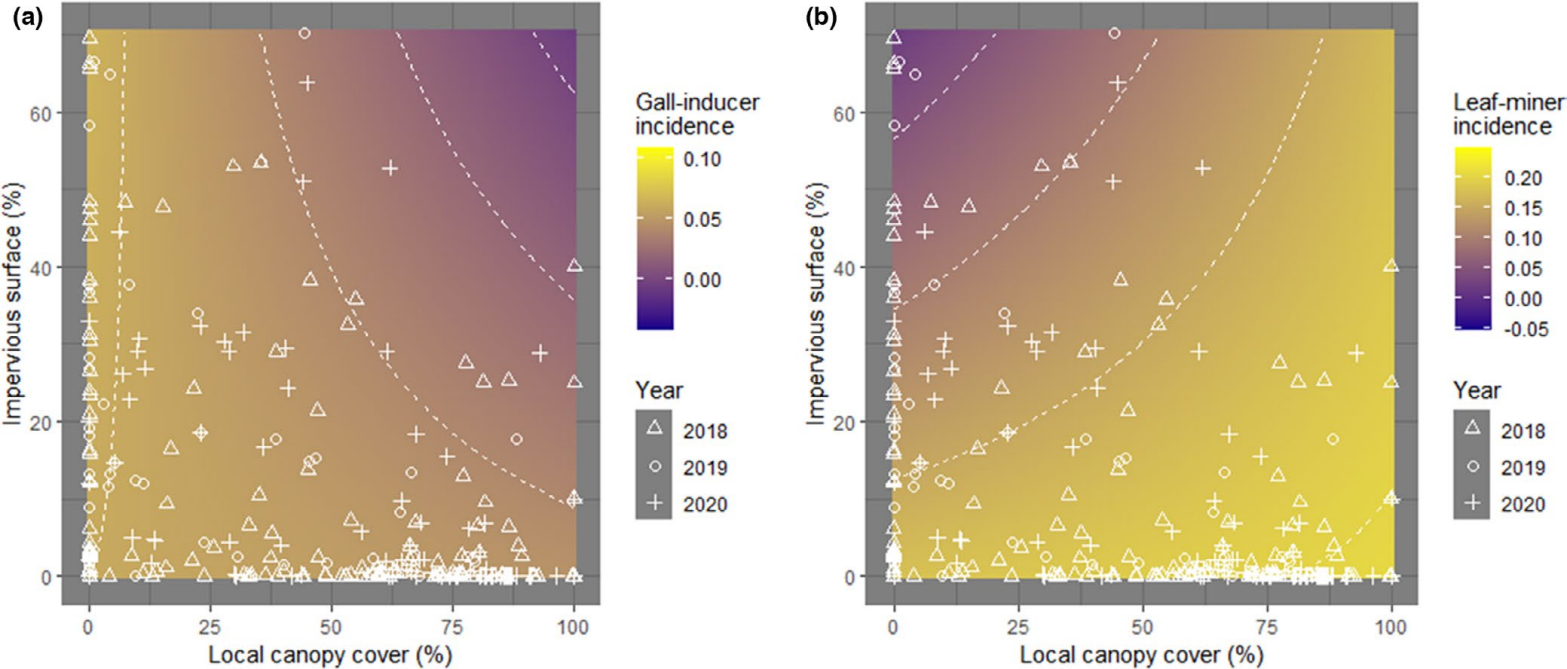
Interactive effect of percentage of impervious surface and of local canopy cover (measured as the cover of impervious surface and local canopy cover within a buffer of 200 and 20 m radius, respectively) on the incidence of gall‐inducing (a) and leaf‐mining herbivores (b)

Insect galls were present on 6.34 ± 0.01% of the inspected leaves. Model selection retained the percentage of impervious surface and local canopy cover, their interaction, year, spring temperature, and spring precipitation as important predictors explaining variability in leaf‐gall incidence (Table S1). The most important variables (RVI = 1) were local canopy cover, sampling year, and average spring temperature (Figure S2B). In particular, gall‐inducer incidence significantly decreased with increasing impervious surface (from 6.30% to 5.00% along the impervious surface range, Figure 2b, Figure S3B) and with increasing local canopy cover (from 8.00% to 0.06% along the range of local canopy cover, Figure 2b, Figure S3E). The effect of impervious surface on gall‐inducer incidence was, however, contingent on local canopy cover (significant impervious surface ×local canopy cover interaction; Figures 2b and 3a): the negative effect of impervious surface on gall‐inducer incidence was more pronounced when there was a greater canopy cover around focal oaks. The incidence of gall inducers was significantly lower in 2019 and 2020 as compared to 2018, and significantly increased with increasing spring temperature (Figure 2b). Spring precipitation had no consistent effect on gall‐inducer incidence (Figure 2b) and also had the lowest relative importance (RVI < 0.60, Figure S2B).

Leaf miners were present in 17.98 ± 0.01% of the sampled leaves. Model selection retained the percentage of impervious surface, local canopy cover, their interaction, year, and spring precipitation as important predictors explaining variability in leaf‐miner incidence (Table S1). The most important variables (RVI = 1) were the percentage of impervious surface, local canopy cover, their interaction, and the sampling year (Figure S2C). Specifically, leaf‐miner incidence significantly decreased with increasing impervious surface (from 21.33% to 1.00% along the range of impervious surface, Figure 2c, Figure S3). The effect of impervious surface on leaf‐miner incidence was, however, contingent on local canopy cover (significant impervious surface ×local canopy cover interaction; Figures 2c and 3b): the negative effect of impervious surface on leaf‐miner incidence was more pronounced when there was a lower canopy cover around focal oaks. It was significantly lower in 2019 and 2020 as compared to 2018 (Figure 2c). Spring precipitation and local canopy cover had no consistent effect on leaf‐miner incidence (Figure 2c, Figure S3) whereas spring temperature was not retained and had the lowest relative importance (RVI < 0.26, Figure S2C).

## 4 DISCUSSION

Our study revealed that impervious surfaces can consistently reduce insect herbivory on the pedunculate oak throughout its geographic range. The effect of impervious surface was partially modulated by the percentage of canopy cover around oaks, with differences among herbivore feeding guilds. Specifically, we found that impervious surface and local canopy cover had independent and opposite effects on overall leaf damage. In contrast, effects of impervious surface on gall‐inducer and leaf‐miner incidence depended on local canopy cover, with a more pronounced negative effect of impervious surface on gall‐inducer and leaf‐miner incidence when there was a greater and lower local canopy cover, respectively. These results show that—just like in non‐urban areas—plant–herbivore interactions in cities are structured by a complex set of interacting factors. This highlights that local habitat characteristics within cities have the potential to attenuate or modify the effect of impervious surfaces on biotic interactions.

### 4.1 Effect of impervious surface on herbivory

The incidence of both gall‐inducing and leaf‐mining herbivores decreased with increasing impervious surface. Given that due to the sampling design of our study, the percentage of impervious surface around oak trees was generally low, it is possible that our findings underestimate the importance of this factor. Thus, we acknowledge that the percentage of impervious surface may not reflect the complexity of urbanization, as some of the oak trees included in this study may have been in green areas embedded within large cities, or in small towns within larger forests. Still, this result aligns with previous reports that have shown that urbanization reduces the abundance and diversity of several guilds of insect herbivores (Barr et al., 2021; Dobrosavljević et al., 2020; Fenoglio et al., 2020; Herrmann et al., 2012; Kozlov et al., 2017; Moreira et al., 2019) with the noticeable exception of sap‐feeding herbivores (de Andrade & Rivkin, 2020; Dale et al., 2016; Parsons & Frank, 2019; Raupp et al., 2010). For instance, Herrmann et al. (2012) found lower species richness of galler communities on the valley oak (*Quercus lobata*) in urban areas than in natural areas. Similarly, Dobrosavljević et al. (2020) found lower richness, abundance, and diversity of leaf‐miner communities on the pedunculate oak in urban areas than in natural areas.

We also found that leaf damage decreased with increasing impervious surface around focal oaks. Although this result aligns with the observation that the incidence of gall‐inducing and leaf‐mining herbivores decreased with increasing impervious surface, it is important to consider that leaf damage (i.e., the amount of biomass consumed by chewing herbivores and/or by leaf miners) may not scale proportionally with the abundance and diversity of insect herbivores. Several other ecological factors that are influenced by impervious surface may determine the amount of damage caused by herbivores. They include the top‐down control of herbivore populations by their enemies (Kozlov et al., 2017; Planillo et al., 2021; Turrini et al., 2016) as well as plant defenses and quality (Moreira et al., 2019; Thompson et al., 2016). The correlative nature of our data does not allow further robust inferences on the mechanisms underlying the observed patterns.

### 4.2 Effect of local canopy cover on herbivory

Herbivory varied with canopy cover in the immediate vicinity of oaks, but this effect was guild specific: there was an independent positive effect of local canopy cover on herbivory, a negative effect on gall‐inducing herbivores, and no effect on leaf‐mining herbivores. Several studies have compared herbivore abundance or diversity, and sometimes herbivory, between urban and forested environments (Herrmann et al., 2012; Kozlov et al., 2017; Moreira et al., 2019), while others have addressed the effect of urban tree density on insect herbivores (Barr et al., 2021; Christie et al., 2010; Christie & Hochuli, 2005; Herrmann et al., 2012; Long & Frank, 2020; Meyer et al., 2020; Raupp et al., 2010). However, findings were contradictory with reports of both higher (Christie & Hochuli, 2005) and lower (Herrmann et al., 2012; Long & Frank, 2020) herbivory in isolated trees as compared to trees growing in larger forest patches. This effect of local canopy cover also mirrors variability in the response of herbivory to the size of forest fragments (De La Vega et al., 2012; Kaartinen & Roslin, 2011; Simonetti et al., 2007; Valdés‐Correcher et al., 2019). We, therefore, refrain from putting forth any particular mechanism that may underlie the patterns we observed. An important reason is that the diversity of herbivores—in particular that of specialist herbivores such as leaf miners and gallers—as well as the damage they cause to a tree are strongly influenced by factors that we could not control in this study, such as the size of the tree and its external appearance, the identity and diversity of oak neighbors, or the distance between focal oaks, other oaks, and non‐oak species or more generally their location within forest patches (Guyot et al., 2019; Jactel et al., 2021; van Schrojenstein Lantman et al., 2018; Smilanich et al., 2016). However, we speculate that denser tree canopies may have buffered microclimatic variations (Coley & Barone, 1996; Dale & Frank, 2014b; Yamasaki & Kikuzawa, 2003; Ziter et al., 2019), which may have been particularly favorable to chewing herbivores that are external feeders (Savilaakso et al., 2009) and at the same time unfavorable to leaf‐galling herbivores which benefit from high temperatures (Valdés‐Correcher et al., 2021; but see Price et al., 1998). Alternatively, top‐down forces also vary with local canopy cover and may consequently influence insect herbivory. For instance, predation activity of birds (Stemmelen et al., 2020) and the abundance of birds (Valdés‐Correcher et al., 2019) increase with increasing local canopy cover. However, if the observed negative association between herbivory and forest cover is mediated by bird predation, the opposite pattern would be expected.

### 4.3 Interactive effect of impervious surface and local canopy cover on herbivory

Canopy cover in cities varies widely. The design of our study allowed us to partially disentangle the response of herbivory to the joint variation in impervious surface and local canopy cover. We found that increasing local canopy cover modulated the effect of impervious surface on some herbivores. Specifically, the negative effect of impervious surface on gall‐inducing herbivores strengthened with increasing local canopy cover, whereas increasing local canopy cover annulled the effect of impervious surface on leaf‐mining herbivores. Impervious surface and local canopy cover have antagonistic effects on the microclimate and enemy pressure. Cities are warmer than the surrounding rural areas as a result of the “heat island effect” (Kalnay & Cai, 2003; Parker, 2010; Roth et al., 1989; Ziter et al., 2019), which is locally buffered by the presence of trees (Loughner et al., 2012; Nuruzzaman, 2015; Ziter et al., 2019). Forest patches in urban environments serve as habitats for both herbivores and predators, which is likely to modify the strength of horizontal (herbivore–herbivore) and vertical (herbivore–predator) interactions in urban trees (Long et al., 2019; Long & Frank, 2020). Endophagous herbivores such as gall inducers and leaf miners are more sheltered from the environment than ectophagous herbivores. Thus, ectophagous herbivores may be more sensitive to local environmental conditions than endophagous herbivores. For instance, we found a positive relationship between the incidence of gall‐inducing herbivores and temperature. It is possible that by buffering the heat island effect, the presence of a denser canopy reduced the incidence of gall inducers on oaks. However, this interpretation needs to be taken with caution because local canopy cover and temperature were not correlated. On the contrary, leaf‐mining herbivores were found to be favored by lower (Gaston et al., 2004) or intermediate temperatures (Valdés‐Correcher et al., 2021). For these herbivores, a denser canopy could have negated the heat island effects, creating more favorable habitats. We cannot exclude that the interactive effect of impervious surface and canopy cover was partially dependent on differential predation rates, but this could not be investigated in the present study.

### 4.4 Effect of climate on insect herbivory

Climatic variables were included in the analyses to take into account that oaks were sampled along a latitudinal gradient. Climate had a significant effect on insect herbivory and this effect varied among feeding guilds. Consistent with previous studies (Kozlov et al., 2016; Valdés‐Correcher et al., 2021), we found that precipitation had a negative effect on leaf damage (Castagneyrol et al., 2018; but see Kozlov et al., 2015), temperature had a positive effect on gall‐inducer incidence (Price et al., 1998), whereas leaf‐miner incidence did not vary with climate (Leckey et al., 2014). The differences in the effect of climate among feeding guilds may be due to differences in insect herbivore strategies to survive different climatic conditions, which was discussed extensively in a previous paper (see Valdés‐Correcher et al., 2021).

## 5 CONCLUSIONS

Our consideration of the effect of impervious surface and local canopy cover on insect herbivory provides novel insights into plant–herbivore interactions. We found that insect herbivory responds simultaneously to both impervious surface and local canopy cover in the pedunculate oak in the major part of its geographic range. Importantly, our results highlight that impervious surface has a negative effect on insect herbivory across the three feeding guilds. However, local canopy cover as well as its interaction with impervious surface influenced insect herbivory of different feeding guilds differently. Thus, local canopy cover within cities has the capacity to mitigate or modify the effect of impervious surface on biotic interactions, as it differentially influences the effect of impervious surface on herbivores. Important insights will be gained by investigating the mechanisms driving these patterns, in particular by deciphering the interactive effects of impervious surface and canopy cover on the microclimate and natural enemy pressures herbivores are exposed to.

## CONFLICT OF INTEREST

The authors declare no competing financial interests.

## AUTHOR CONTRIBUTIONS

**Elena Valdés‐Correcher:** Conceptualization (lead); Data curation (lead); Formal analysis (lead); Investigation (lead); Methodology (lead); Project administration (lead); Supervision (equal); Validation (lead); Visualization (lead); Writing – original draft (lead). **Anna Popova:** Methodology (equal); Validation (equal). **Andrea Galmán:** Investigation (equal); Methodology (equal); Validation (equal). **Andreas Prinzing:** Investigation (equal); Methodology (equal); Validation (equal). **Andrey V. Selikhovkin:** Methodology (equal); Validation (equal). **Andy G. Howe:** Investigation (equal); Methodology (equal); Validation (equal). **Anna Mrazova:** Methodology (equal); Validation (equal). **Anne‐Maïmiti Dulaurent:** Investigation (equal); Methodology (equal); Validation (equal). **Arndt Hampe:** Investigation (equal); Validation (equal). **Ayco Jerome Michel Tack:** Investigation (equal); Methodology (equal); Validation (equal). **Christophe Bouget:** Methodology (equal); Validation (equal). **Daniela Lupaștean:** Methodology (equal); Validation (equal). **Deborah Harvey:** Investigation (equal); Methodology (equal); Validation (equal). **Dmitry L. Musolin:** Investigation (equal); Methodology (equal); Validation (equal). **Gábor L. Lövei:** Investigation (equal); Methodology (equal); Validation (equal). **Giada Centenaro:** Methodology (equal); Validation (equal). **Inge Van Halder:** Investigation (equal); Methodology (equal); Validation (equal). **Jonas Hagge:** Investigation (equal); Methodology (equal); Validation (equal). **Jovan Dobrosavljević:** Investigation (equal); Methodology (equal); Validation (equal). **Juha‐Matti Pitkänen:** Methodology (equal); Validation (equal). **Julia Koricheva:** Investigation (equal); Methodology (equal); Validation (equal). **Katerina Sam:** Investigation (equal); Methodology (equal); Validation (equal). **Luc Barbaro:** Investigation (equal); Methodology (equal); Validation (equal). **Manuela Branco:** Investigation (equal); Methodology (equal); Validation (equal). **Marco Ferrante:** Investigation (equal); Methodology (equal); Validation (equal). **Maria Faticov:** Investigation (equal); Methodology (equal); Validation (equal). **Markéta Tahadlová:** Methodology (equal); Validation (equal). **Martin Gossner:** Investigation (equal); Methodology (equal); Validation (equal). **Maxime Cauchoix:** Investigation (equal); Methodology (equal); Validation (equal). **Michał Bogdziewicz:** Investigation (equal); Methodology (equal); Validation (equal). **Mihai‐Leonard Duduman:** Methodology (equal); Validation (equal). **Mikhail V. Kozlov:** Investigation (equal); Methodology (equal); Validation (equal). **Mona C. Bjoern:** Investigation (equal); Methodology (equal); Validation (equal). **Nikita A. Mamaev:** Methodology (equal); Validation (equal). **Pilar Fernandez‐Conradi:** Investigation (equal); Methodology (equal); Validation (equal). **Rebecca L. Thomas:** Methodology (equal); Validation (equal). **Ross Wetherbee:** Investigation (equal); Methodology (equal); Validation (equal). **Samantha Green:** Methodology (equal); Validation (equal). **Slobodan Milanović:** Methodology (equal); Validation (equal). **Xoaquín Moreira:** Investigation (equal); Methodology (equal); Validation (equal). **Yannick Mellerin:** Methodology (equal); Validation (equal). **Yasmine Kadiri:** Methodology (equal); Validation (equal). **Bastien Castagneyrol:** Conceptualization (lead); Data curation (equal); Formal analysis (lead); Funding acquisition (lead); Investigation (lead); Methodology (lead); Project administration (lead); Supervision (lead); Validation (lead); Visualization (lead); Writing – original draft (lead).

## Supporting information

Figure S1

Figure S2

Figure S3

Table S1

Supplementary Material
Supinfo

## Data Availability

The data sets supporting this article are available via an open‐access repository (https://doi.org/10.5061/dryad.k3j9kd59b).
