# Supplementary material for: Herbivory on the pedunculate oak along an urbanization gradient in Europe: Effects of impervious surface, local tree cover, and insect feeding guild
Source: Ecol Evol. 2022 Mar 14;12(3):e8709. doi: 10.1002/ece3.8709 (PMC8928871; doi:10.1002/ece3.8709)
Supplement: Supplementary file 1 — Figure S1 [file ECE3-12-e8709-s004.docx]

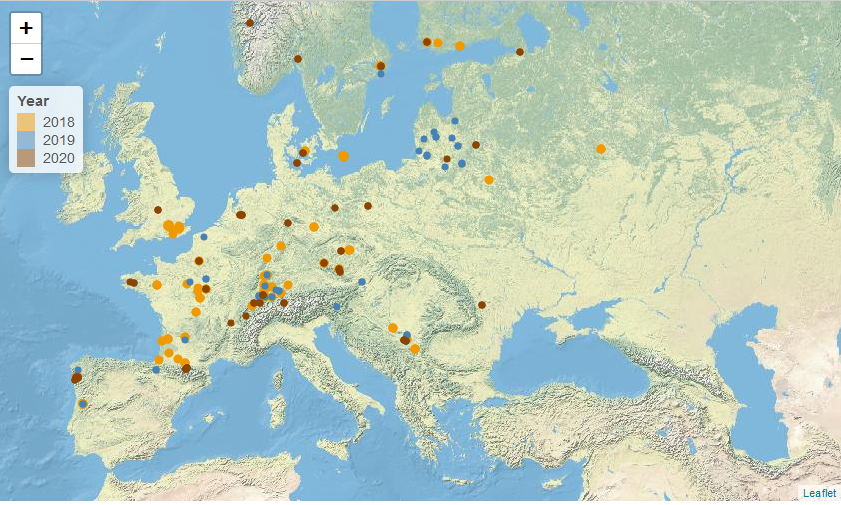


<file:///C:/Users/Elena/SPONFOREST/2021/Paper/Tree%20bodyguards%20URBANIZATION/Ecology%20and%20Evolution/Figure%20A%20appendix.html>
