# Supplementary material for: Herbivory on the pedunculate oak along an urbanization gradient in Europe: Effects of impervious surface, local tree cover, and insect feeding guild
Source: Ecol Evol. 2022 Mar 14;12(3):e8709. doi: 10.1002/ece3.8709 (PMC8928871; doi:10.1002/ece3.8709)
Supplement: Supplementary file 2 — Figure S2 [file ECE3-12-e8709-s003.docx]

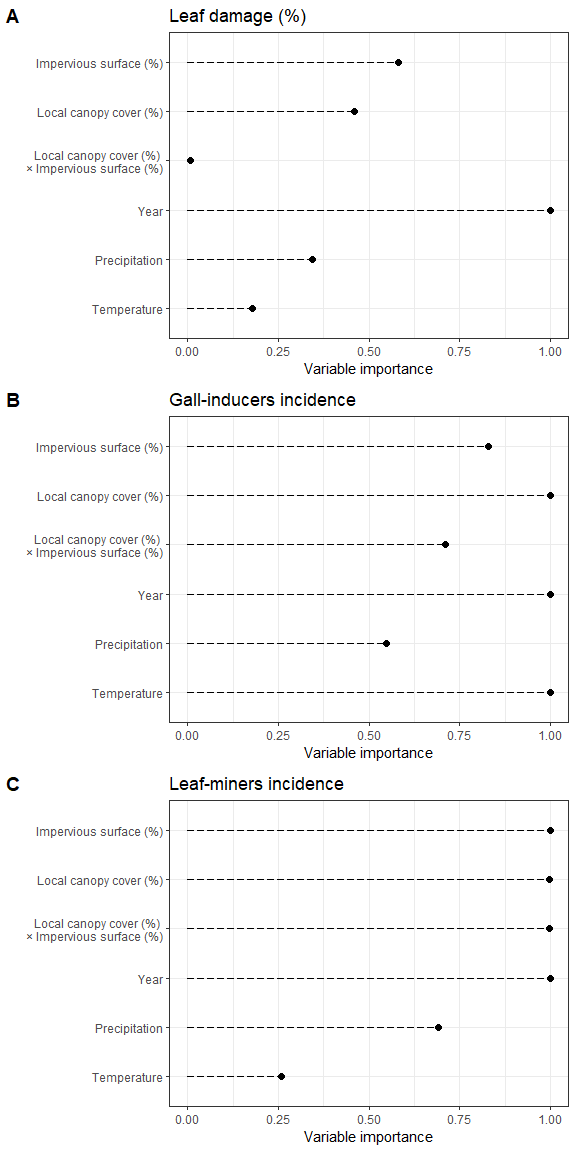


**Figure B**. Relative importance of every variable (RVI) included in the models that considered the effect of percentage of impervious surface and local canopy cover, their interaction, year, mean spring temperature and/or mean spring precipitation (n = 298) on leaf damage (A) and the incidence of gall-inducing (B) and leaf-mining (C) herbivores.
