# Supplementary material for: Herbivory on the pedunculate oak along an urbanization gradient in Europe: Effects of impervious surface, local tree cover, and insect feeding guild
Source: Ecol Evol. 2022 Mar 14;12(3):e8709. doi: 10.1002/ece3.8709 (PMC8928871; doi:10.1002/ece3.8709)
Supplement: Supplementary file 3 — Figure S3 [file ECE3-12-e8709-s002.docx]

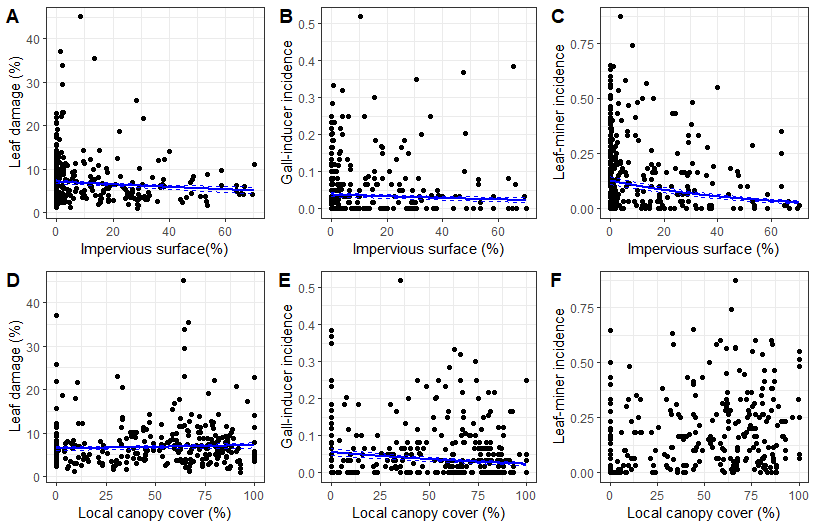


**Figure C**. Effect of impervious surface and of local canopy cover (measured as the cover of impervious surface and local canopy cover within a buffer of 200 and 20 m radius, respectively) on the percentage of leaf damage (A, D), on gall-inducer incidence (B, E) and on leaf miner incidence (C, F) (n = 298).
