# Supplementary material for: Herbivory on the pedunculate oak along an urbanization gradient in Europe: Effects of impervious surface, local tree cover, and insect feeding guild
Source: Ecol Evol. 2022 Mar 14;12(3):e8709. doi: 10.1002/ece3.8709 (PMC8928871; doi:10.1002/ece3.8709)
Supplement: Supplementary file 4 — Table S1 [file ECE3-12-e8709-s005.docx]

**Table A.** Summary of model coefficient parameter estimates (i.e., effect sizes), degrees of freedom (df), log-likelihood, AICc, ΔAICc, AICc weight (wi) and the variance explained by fixed (R2m) and fixed plus random factors (R2c) of the different climatic models. The gradient of colours from red to green corresponds to the effect size, from large negative (red) to large positive (green) effect sizes. The set of models competing with the best model within 2 units of ΔAICc is highlighted in bold font. Year is the effect of each year (2019 and 2020 contrasted with 2018).

| **Models of leaf damage** | | | | | | | | | | | | | |
| --- | --- | --- | --- | --- | --- | --- | --- | --- | --- | --- | --- | --- | --- |
| Intercept | Local canopy  cover | Impervious surface | Local canopy cover  x  Impervious surface | Precipitation | Temperature | Year | df | logLik | | AICc | ΔAICc | W_i_ | *R²m (R²c)* |
| ***1.980*** |  | ***-0.106*** |  |  |  | ***+*** | ***6*** | ***-163.25*** | | ***338.78*** | ***0.00*** | ***0.231*** | ***0.23 (0.63)*** |
| 1.978 | 0.092 |  |  |  |  | + | 6 | -163.59 | | 339.46 | 0.68 | 0.164 | 0.21 (0.63) |
| 1.984 |  | -0.100 |  | -0.092 |  | + | 7 | -162.82 | | 340.02 | 1.24 | 0.124 | 0.26 (0.63) |
| 1.983 | 0.087 |  |  | -0.094 |  | + | 7 | -163.10 | | 340.58 | 1.80 | 0.094 | 0.25 (0.63) |
| 1.992 | 0.069 | -0.081 |  |  |  | + | 7 | -163.24 | | 340.86 | 2.09 | 0.081 | 0.23 (0.62) |
| 1.985 |  | -0.106 |  |  | -0.088 | + | 7 | -163.56 | | 341.49 | 2.72 | 0.059 | 0.24 (0.62) |
| 1.952 |  |  |  |  |  | + | 5 | -165.89 | | 341.99 | 3.22 | 0.046 | 0.21 (0.66) |
| 1.960 |  |  |  | -0.104 |  | + | 6 | -164.93 | | 342.15 | 3.38 | 0.043 | 0.25 (0.65) |
| 1.983 | 0.091 |  |  |  | -0.087 | + | 7 | -163.98 | | 342.33 | 3.56 | 0.039 | 0.23 (0.63) |
| 1.995 | 0.066 | -0.076 |  | -0.087 |  | + | 8 | -163.04 | | 342.57 | 3.80 | 0.035 | 0.26 (0.62) |
| 1.987 |  | -0.099 |  | -0.083 | -0.076 | + | 8 | -163.58 | | 343.65 | 4.88 | 0.020 | 0.27 (0.63) |
| 1.997 | 0.068 | -0.081 |  |  | -0.086 | + | 8 | -163.60 | | 343.69 | 4.91 | 0.020 | 0.25 (0.61) |
| 1.986 | 0.086 |  |  | -0.086 | -0.075 | + | 8 | -163.92 | | 344.34 | 5.56 | 0.014 | 0.26 (0.63) |
| 1.957 |  |  |  |  | -0.090 | + | 6 | -166.26 | | 344.81 | 6.04 | 0.011 | 0.22 (0.65) |
| 1.963 |  |  |  | -0.097 | -0.078 | + | 7 | -165.70 | | 345.79 | 7.01 | 0.007 | 0.26 (0.65) |
| 1.998 | 0.065 | -0.076 |  | -0.079 | -0.074 | + | 9 | -163.85 | | 346.32 | 7.54 | 0.005 | 0.27 (0.61) |
| 1.984 | 0.066 | -0.103 | -0.031 |  |  | + | 8 | -165.32 | | 347.13 | 8.36 | 0.004 | 0.24 (0.62) |
| 1.986 | 0.061 | -0.103 | -0.038 | -0.091 |  | + | 9 | -164.90 | | 348.41 | 9.63 | 0.002 | 0.27 (0.61) |
| 1.988 | 0.064 | -0.104 | -0.032 |  | -0.086 | + | 9 | -165.64 | | 349.89 | 11.11 | 0.001 | 0.25 (0.61) |
| 1.989 | 0.060 | -0.102 | -0.038 | -0.082 | -0.073 | + | 10 | -165.71 | | 352.17 | 13.39 | 0.000 | 0.28 (0.61) |
| **Models of leaf-gall incidence** | | | | | | | | | | | | | |
| Intercept | Local canopy  cover | Impervious surface | Local canopy cover  x  Impervious surface | Precipitation | Temperature | Year | df | logLik | | AICc | ΔAICc | W_i_ | *R²m (R²c)* |
| ***-2.571*** | ***-0.263*** | ***-0.220*** | ***-0.150*** | ***0.143*** | ***0.466*** | ***+*** | ***9*** | ***-455.79*** | | ***930.20*** | ***0.00*** | ***0.382*** | ***0.45 (0.86)*** |
| -2.558 | -0.271 | -0.210 | -0.157 |  | 0.467 | + | 8 | -457.01 | | 930.51 | 0.31 | 0.328 | 0.45 (0.86) |
| -2.542 | -0.200 |  |  | 0.140 | 0.468 | + | 7 | -459.33 | | 933.05 | 2.85 | 0.092 | 0.44 (0.85) |
| -2.525 | -0.211 |  |  |  | 0.470 | + | 6 | -460.55 | | 933.38 | 3.18 | 0.078 | 0.44 (0.85) |
| -2.530 | -0.226 | -0.085 |  | 0.158 | 0.466 | + | 8 | -458.50 | | 933.49 | 3.30 | 0.074 | 0.45 (0.85) |
| -2.514 | -0.233 | -0.066 |  |  | 0.469 | + | 7 | -460.02 | | 934.43 | 4.23 | 0.046 | 0.45 (0.85) |
| -2.499 |  |  |  | 0.185 | 0.434 | + | 6 | -466.60 | | 945.48 | 15.28 | 0.000 | 0.44 (0.84) |
| -2.537 | -0.251 | -0.226 | -0.157 | 0.139 |  | + | 8 | -465.35 | | 947.18 | 16.99 | 0.000 | 0.35 (0.84) |
| -2.531 | -0.261 | -0.217 | -0.166 |  |  | + | 7 | -466.48 | | 947.34 | 17.15 | 0.000 | 0.36 (0.85) |
| -2.502 |  | 0.012 |  | 0.182 | 0.435 | + | 7 | -466.58 | | 947.53 | 17.34 | 0.000 | 0.44 (0.84) |
| -2.475 |  |  |  |  | 0.438 | + | 5 | -468.87 | | 947.94 | 17.74 | 0.000 | 0.44 (0.84) |
| -2.484 |  | 0.036 |  |  | 0.439 | + | 6 | -468.67 | | 949.63 | 19.43 | 0.000 | 0.43 (0.84) |
| -2.507 | -0.186 |  |  | 0.138 |  | + | 6 | -469.04 | | 950.37 | 20.17 | 0.000 | 0.34 (0.84) |
| -2.496 | -0.199 |  |  |  |  | + | 5 | -470.21 | | 950.62 | 20.42 | 0.000 | 0.34 (0.84) |
| -2.494 | -0.213 | -0.085 |  | 0.158 |  | + | 7 | -468.23 | | 950.84 | 20.64 | 0.000 | 0.34 (0.84) |
| -2.485 | -0.221 | -0.065 |  |  |  | + | 6 | -469.72 | | 951.73 | 21.54 | 0.000 | 0.35 (0.84) |
| -2.472 |  |  |  | 0.186 |  | + | 5 | -475.33 | | 960.87 | 30.67 | 0.000 | 0.34 (0.83) |
| -2.474 |  | 0.011 |  | 0.183 |  | + | 6 | -475.32 | | 962.92 | 32.72 | 0.000 | 0.34 (0.83) |
| -2.452 |  |  |  |  |  | + | 4 | -477.54 | | 963.22 | 33.02 | 0.000 | 0.34 (0.84) |
| -2.462 |  | 0.038 |  |  |  | + | 5 | -477.34 | | 964.89 | 34.69 | 0.000 | 0.34 (0.84) |
| **Models of leaf-miner incidence** | | | | | | | | | | | | | |
| Intercept | Local canopy  cover | Impervious surface | Local canopy cover  x  Impervious surface | Precipitation | Temperature | Year | df | logLik | | AICc | ΔAICc | W_i_ | *R²m (R²c)* |
| ***-1.742*** | ***0.079*** | ***-0.323*** | ***0.178*** | ***0.159*** |  | ***+*** | ***8*** | ***-680.75*** | | ***1377.99*** | ***0.00*** | ***0.512*** | ***0.20 (0.93)*** |
| -1.718 | 0.078 | -0.311 | 0.170 |  |  | + | 7 | -682.62 | | 1379.62 | 1.63 | 0.227 | 0.21 (0.92) |
| -1.742 | 0.079 | -0.323 | 0.178 | 0.160 | 0.014 | + | 9 | -680.74 | | 1380.10 | 2.11 | 0.178 | 0.21 (0.93) |
| -1.719 | 0.079 | -0.311 | 0.170 |  | -0.014 | + | 8 | -682.61 | | 1381.71 | 3.72 | 0.080 | 0.21 (0.92) |
| -1.801 |  | -0.423 |  | 0.132 |  | + | 6 | -689.37 | | 1391.03 | 13.04 | 0.001 | 0.17 (0.93) |
| -1.779 |  | -0.410 |  |  |  | + | 5 | -690.67 | 1391.54 | | 13.55 | 0.001 | 0.18 (0.92) |
| -1.789 | 0.041 | -0.404 |  | 0.130 |  | + | 7 | -688.85 | 1392.08 | | 14.09 | 0.000 | 0.18 (0.93) |
| -1.767 | 0.041 | -0.391 |  |  |  | + | 6 | -690.12 | 1392.53 | | 14.54 | 0.000 | 0.19 (0.92) |
| -1.801 |  | -0.423 |  | 0.133 | 0.010 | + | 7 | -689.37 | 1393.12 | | 15.13 | 0.000 | 0.17 (0.93) |
| -1.780 |  | -0.410 |  |  | -0.015 | + | 6 | -690.66 | 1393.60 | | 15.61 | 0.000 | 0.18 (0.92) |
| -1.789 | 0.041 | -0.404 |  | 0.130 | -0.001 | + | 8 | -688.85 | 1394.19 | | 16.20 | 0.000 | 0.18 (0.93) |
| -1.768 | 0.042 | -0.391 |  |  | -0.024 | + | 7 | -690.09 | 1394.56 | | 16.58 | 0.000 | 0.18 (0.92) |
| -2.100 | 0.024 | -0.312 | 0.181 | 0.277 |  |  | 6 | -712.58 | 1437.45 | | 59.46 | 0.000 | 0.11 (0.94) |
| -2.101 | 0.021 | -0.315 | 0.182 | 0.293 | 0.086 |  | 7 | -712.29 | 1438.97 | | 60.98 | 0.000 | 0.12 (0.94) |
| -1.851 | 0.137 |  |  |  |  | + | 5 | -717.51 | 1445.22 | | 67.23 | 0.000 | 0.08 (0.92) |
| -2.077 | 0.020 | -0.292 | 0.168 |  |  |  | 5 | -717.78 | 1445.76 | | 67.77 | 0.000 | 0.08 (0.93) |
| -1.852 | 0.139 |  |  |  | -0.076 | + | 6 | -717.24 | 1446.77 | | 68.78 | 0.000 | 0.09 (0.92) |
| -1.860 | 0.138 |  |  | 0.053 |  | + | 6 | -717.29 | 1446.87 | | 68.88 | 0.000 | 0.08 (0.92) |
| -2.077 | 0.020 | -0.292 | 0.168 |  | 0.008 |  | 6 | -717.77 | 1447.83 | | 69.85 | 0.000 | 0.08 (0.93) |
| -1.859 | 0.140 |  |  | 0.046 | -0.067 | + | 7 | -717.08 | 1448.55 | | 70.56 | 0.000 | 0.08 (0.92) |
