# Supplementary material for: Herbivory on the pedunculate oak along an urbanization gradient in Europe: Effects of impervious surface, local tree cover, and insect feeding guild
Source: Ecol Evol. 2022 Mar 14;12(3):e8709. doi: 10.1002/ece3.8709 (PMC8928871; doi:10.1002/ece3.8709)
Supplement: Supplementary file 5 — Supplementary Material Supinfo [file ECE3-12-e8709-s001.docx]

**Figure S1**. Interactive version of the map (Figure 1A) showing the location of trees sampled in 2018 (yellow circles), 2019 (blue circles) and 2020 (brown circles) by scientists and partner schools.

**Figure S2**. Relative importance of every variable (RVI) included in the models that considered the effect of percentage of impervious surface and local canopy cover, their interaction, year, mean spring temperature and/or mean spring precipitation (n = 298) on leaf damage (A) and the incidence of gall-inducing (B) and leaf-mining (C) herbivores.

**Figure S3**. Effect of impervious surface and of local canopy cover (measured as the percentage of impervious surface and local canopy cover within a buffer of 200 and 20 m radius, respectively) on the percentage of leaf damage (A, D), on gall-inducer incidence (B, E) and on leaf miner incidence (C, F) (n = 298).

**Table S1.** Summary of model coefficient parameter estimates (i.e., effect sizes), degrees of freedom (df), log-likelihood, AICc, ΔAICc, AICc weight (wi) and the variance explained by fixed (R2m) and fixed plus random factors (R2c) of the different climatic models. The gradient of colours from red to green corresponds to the effect size, from large negative (red) to large positive (green) effect sizes. The set of models competing with the best model within 2 units of ΔAICc is highlighted in bold font. Year is the effect of each year (2019 and 2020 contrasted with 2018).
